# Excessive mechanical stress-induced intervertebral disc degeneration is related to Piezo1 overexpression triggering the imbalance of autophagy/apoptosis in human nucleus pulpous

**DOI:** 10.1186/s13075-022-02804-y

**Published:** 2022-05-23

**Authors:** Sheng Shi, Xing-Jian Kang, Zhi Zhou, Zhi-Min He, Shuang Zheng, Shi-Sheng He

**Affiliations:** 1 Department of Orthopaedics, Shanghai Tenth People’s Hospital, Tongji University, Shanghai, 200072 People’s Republic of China; 2 Spinal Pain Research Institute, Tongji University School of Medicine, Shanghai, 200072 People’s Republic of China; 3 School of Stomatology, Wenzhou Medical University, Wenzhou, 325000 People’s Republic of China; 4 School of Medicine, Shanghai University, Shanghai, 200444 People’s Republic of China

**Keywords:** Intervertebral disc degeneration, Mechanical stress, Piezo 1, Nucleus pulpous, Autophagy

## Abstract

**Background:**

Mechanical stress plays a crucial role in the pathogenesis of intervertebral disc degeneration (IVDD). The mechanosensitive Piezo1 ion channel can sense the changes in mechanical stress and convert the mechanical signals into chemical signals. This study aims to investigate the effect of Piezo1 on the mechanical stress-induced IVDD and explore the possible mechanism.

**Methods:**

The expression of Piezo1 and collagen II in immunohistochemical staining, cervical curvature, and the stiffness of nucleus pulpous (NP) were performed in normal and degenerated human intervertebral discs. In the experiment, high-intensity compression was applied to mimic the mechanical environment of IVDD. The cell viability, matrix macromolecules, and pro-inflammatory cytokines were examined to investigate the effect of Piezo1 on mechanical stress-treated NP cells. Additionally, autophagy condition of NP cells was detected within high-intensity compression and/or the inhibitor of Piezo1, GsMTx4.

**Results:**

The up-expression of Piezo1, down-expression of Col II, elevated stiffness of NP, and poor kyphosis were observed in degenerated human intervertebral discs. High-intensity stress significantly decreased cell viability and the synthesis of extracellular matrix but increased the expression of senescence-associated proteins (p53 and p16) and pro-inflammatory cytokines (TNF-α, IL-6 and IL-1β) by mitochondrial dysfunction and suppression of autophagy. However, GsMTx4 can partly attenuate these effects.

**Conclusion:**

Piezo1 upregulation under excessive mechanical stress promotes the apoptosis, senescence, and pro-inflammatory cytokines of NP and leads to the loss of extracellular matrix by mitochondrial dysfunction and the suppression of autophagy; on the other hand, the inhibition of Piezo1 can partly alleviate these effects.

## Introduction

Intervertebral disc degeneration (IVDD) is the leading cause of degenerative spine diseases such as discogenic pain and disc herniation, which reduces the quality of life and increases the socioeconomical burden. However, the onset and development of IVDD is a complex process involving numerous factors such as biomechanics, aging, genetic factors, nutrition, inflammation, and so on. Among the aforementioned factors, mechanical stress as part of the biomechanics factors may contribute to playing a particularly prominent role in the pathogenesis of IVDD [1–5].

The nucleus pulposus (NP), localized in the center of intervertebral disc, is considered to be the most crucial component for maintaining the pressure gradient and ensuring the infiltration and diffusion of metabolites and nutrients. However, abnormal mechanical load can promote cell death in the NP, cause matrix metabolism disorders, and hasten the process of IVDD. It is suggested that compressive forces by bad postures or kyphosis may lead to intense stresses that act on the NP. Furthermore, mechanical stress caused by kyphosis has been linked to the pathological process of IVDD [6]. However, the molecular mechanism by which the NP cells were affected and produced pro-inflammatory mediators in response to abnormal load needs to be investigated further. Piezo1 has been confirmed to be a new mechanosensitive ion channel [7], which can sense the changes of mechanical stress and convert the mechanical signals into electrical or chemical signals. It is widely distributed in a variety of different tissues including bone, articular cartilage and the intervertebral disc [8–13].

In the current study, we aim to assess whether Piezo1 can be activated in the NP by abnormal mechanical stress, observe the effect of Piezo1 on the mechanical stress-induced IVDD, and investigate the possible mechanism.

## Materials and methods

### Tissue samples collection

Cervical NP samples were collected through anterior cervical decompression surgery from 20 patients (eight men and twelve women; age range: 35–65 years) with cervical myelopathy or radiculopathy due to soft disc herniation and six patients (four men and two women; age range: 22–32 years) with cervical fracture or acute cervical disc herniation refractory to conservative treatment. The study protocol was approved by the Ethics Committee of Shanghai Tenth People’s Hospital Affiliated to Tongji University (SHSY-IEC-21KN45). Written informed consent was obtained from all patients.

### Radiological assessment and NP stiffness

The degree of intervertebral disc degeneration was evaluated preoperatively via the Pfirrmann grade system (grades I–V) according to magnetic resonance imaging (MRI) [14]. Segmental angle was estimated by measuring the angle between the superior endplate of the rostral vertebra involved and the inferior endplate of the caudal vertebra involved on the lateral radiograph [15]. Lordosis was expressed as a positive value, while kyphosis was expressed as a negative value. All the data of MRI and radiograph were independently measured by the experienced radiologist and surgeons. Surface stiffness was tested with an atomic force microscope (AFM, INNOVA, Bruker Nano, Inc., USA) at the center of the human NP. The elastic modulus was calculated according to a previous study [16].

### Isolation and culture of NPs

Nucleus pulposus (NP) tissues from appropriately normal group were collected and cut into fragments and then digested with 0.25% trypsin (Corning, USA) for 40 min and 2 g/L type II collagenase (Roche, Tokyo, Japan) for 5 h at 37 °C. The tissue digests were filtered through a 75-μm membrane filtration and transferred to a 15-ml conical tube for cell collection. Cells were cultured in DMEM/F-12 medium (Gibco, Grand Island, NY, USA) containing 15% fetal bovine serum (FBS) and streptomycin (0.1 mg/ml) and penicillin (100 U/ml) (Invitrogen, Carlsbad, CA, USA) at 37 °C and 5% CO_2_.

### Application of mechanical stress

When cells had grown to 80% confluence, they were detached using 0.25% trypsin-EDTA and then suspended in mixed medium containing DMEM/F-12 and 4% low melting point agarose (Sigma, Germany). The mixture was cultured in 6-well BioFlex plates, and the compression experiments were conducted with Flexcell FX5000 Compression system (Flexcell International, McKeesport, USA). The frequency was set to 1.0 Hz with 15% or 1.5% compression intensity, and the cells were gathered after 12 h, 24 h, and 48 h, respectively. The control group was cultured under the same conditions without exposure to mechanical stress. When Piezo1 inhibitor GsMTx4 was used on NP cells, the cells were pre-incubated with GsMTx4 (2.5 μM) 1 h before administration of mechanical stress (15% compression intensity, 48 h).

### Hematoxylin and eosin staining

Intervertebral disc tissues were fixed in 4% paraformaldehyde and embedded in paraffin for sectioning. After the sections were deparaffinized with xylene and ethanol, they were stained with hematoxylin and eosin (H&E) (G1120, Solarbio). Pathological changes were observed under a microscope.

### Immunohistochemistry staining

For the immunohistochemistry staining, human intervertebral disc tissues were fixed with 4% paraformaldehyde, embedded in paraffin, and then processed by routine procedures. After deparaffinization, rehydration, antigen retrieval, and blocking, the slides were incubated with primary antibodies against Piezo1 (No. NBP1-78537, Novus Biologicals, USA, 1:100 dilution) and type II Collagen (No. NBP1-77795, Novus Biologicals, USA, 1:200 dilution) on a humidified box at 4 °C overnight. Then, sections were incubated with secondary antibody for 1 h. DAB substrate kit was used for the color-reaction, and hematoxylin was used for nucleus counterstaining. The results were obtained with a microscope (Olympus, Tokyo, Japan).

### Cell viability

Cell viability assays was performed using MTT cell viability/cytotoxicity assay kit (Beyotime Biotechnology, China) according to manufacturer’s protocol. The cells were treated with different compression intensity of mechanical stress for different lengths of time (0, 12, 24, 48 h). The control and stressed cells were planted into 96-well culture plates. MTT solution was added to the cells after cell plantation. After incubated with MTT solution for 4 h and Formazan solvent for 3 h, the absorbance was measured by OD at 570 nm using a microplate reader.

### Quantitative real-time PCR

The total RNA was extracted from NP tissues using TRIzol reagent (Invitrogen, USA), and the process of RNA reverse transcription was accomplished with PrimeScript RT reagent Kit (TaKaRa, Osaka, Japan). Quantitative real-time PCR was performed using a SYBR Green Premix Ex Taq (Takara, Osaka, Japan) in order to quantify the transcripts of target genes on a thermocycler (LightCycler 480, Roche Applied Science, Mannheim). Amplification conditions were as follows: 45 cycles at 95 °C for 30 s, 95 °C for 10 s, and 60 °C for 20 s. Relative gene expression was determined using the 2^−△△Ct^ method and normalized to β-actin in the same sample. The primers were listed in Table 1.

**Table 1:** Primers for real-time PCR

Target gene	Primers pairs
β-actin	Forward 5’-CCTGTACGCCAACACAGTGC-3’ Reverse 5’-ATACTCCTGCTTGCTGATCC-3’
Piezo1	Forward 5’- GAGATGATGGACAGAGAC-3’ Reverse 5’-AGTAATGGCTAAGGAAGAC -3’
p53	Forward 5’-AGAGCTGAATGAGGCCTTGGAA-3’ Reverse 5’-GAGTCAGGCCCTTCTGTCTTGAAC-3’
p16	Forward 5’-CGGTCGTACCCCGATTCAG-3’ Reverse 5’- GCACCGTAGTTGAGCAGAAGAG-3’

### Western blot analysis

Proteins were extracted and the protein levels were determined by BCA assay (Thermo Scientific, Waltham, MA, USA). Equivalent amounts of proteins were separated by SDS-PAGE and transferred to nitrocellulose filter (NC) membranes for immunoblotting. The blots were probed overnight with primary antibodies against Piezo1 (Novus Biologicals, USA), collagen II, aggrecan (from Abcam, USA), p53, p16, LC3, Beclin-1, p62, Bax, Bcl-2, and β-actin (from Cell Signaling Technology, USA), followed by incubation with fluorescently-labeled secondary antibody (LI-COR, USA). Labeled protein bands were detected by odyssey imaging system and quantified with Image J software (Rawak Software, Inc. Germany).

### Immunofluorescence staining

For immunofluorescence staining of Piezo1 and LC3, NP cells were seeded on coverslips of 6-well microtiter plates and after appropriate treatments; they were incubated with anti-Piezo1 and anti-LC3 antibody (Cell Signaling Technology, USA) at optimal working concentration (1:200) overnight and then stained with a secondary antibody for 1 h. Finally, the nuclei were stained with DAPI for 5 min. Images were obtained with a confocal laser scanning microscopy (Leica microsystem, Germany) and fluorescence intensity was measured by Image J software.

### ELISA

The concentrations of pre-inflammatory cytokines TNF-α, IL-6, and IL-1β in the cell supernatant were measured by commercial ELISA kits (R&D Systems, Minneapolis, MN) according to the manufacturer’s instructions.

### Mitochondrial membrane potential assay

The mitochondrial membrane potential (MMP) was measured by JC-1 probe. After treatment with mechanical stress and/or GsMTx4, the cells were treated with the JC-1 probe according to the manufacturer’s instructions. A flow cytometer was used for analysis. The MMP of each sample was calculated by dividing the ratio of red fluorescence intensity by that of green fluorescence intensity.

### Oxygen consumption rate

The oxygen consumption rate (OCR) of normal and stress-administrated (15% compression intensity, 48 h) NP cells were measured by Seahorse XFe96 Extracellular Flux Analyzer at basal conditions and with serial administration of 1 μM oligomycin, 300 nM carbonyl cyanide-4-(trifluoromethoxy) phenylhydrazone (FCCP) and 1 μM rotenone. OCR was calculated and normalized to protein amount per well.

### Flow cytometry

The apoptosis rate in human NP cells was assessed using an Annexin V-FITC/PI apoptosis detection kit (BD Biosciences, USA) following the manufacturer’s protocols. The fluorescence intensities were analyzed by Flow Cytometry (BD, FACS Canto II).

### Statistical analysis

Data were expressed as mean ± SD. Statistical differences were determined by Student’s *t* test or ANOVA followed by multiple comparisons test. All statistical analyses were performed using SPSS 19.0 (SPSS Inc., Chicago, IL, USA). *P* < 0.05 was considered statistically significant.

## Results

### The upregulated expression of Piezo1, poor curvature, and increased extracellular matrix (ECM) stiffness in the degenerated human NP

**Fig. 1:**
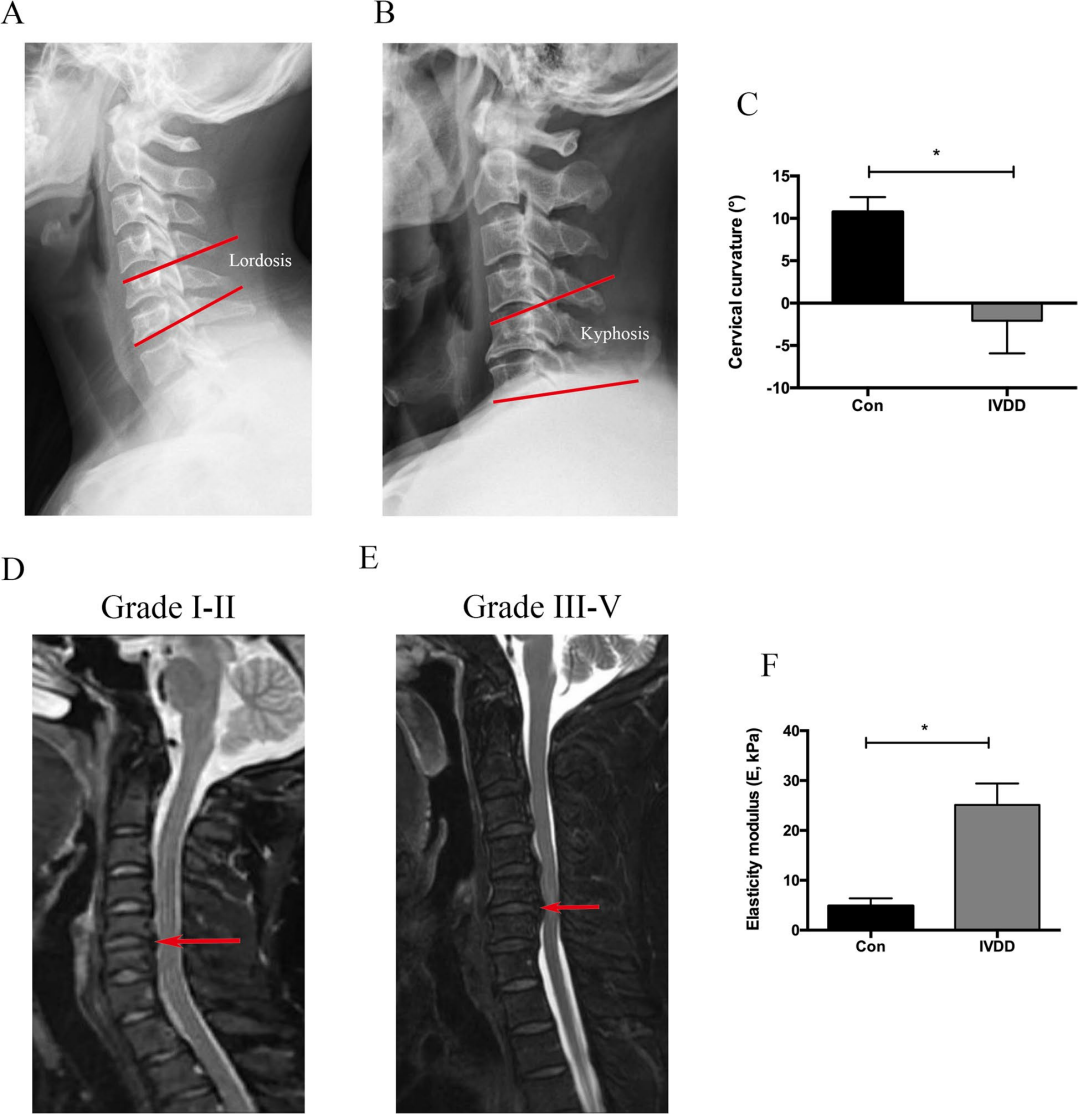
The difference of curvature and elasticity modulus between different Pfirrmann grade groups. **A**, **D** The involved disc shows focal lordosis in the low Pfirrmann grade group. **B**, **E** The involved disc shows focal lordosis in the high Pfirrmann grade group. **C** The comparison of cervical curvature between the two groups. **F** The comparison of elasticity modulus between the two groups. *p < 0.05

It was found that the protein expression of Piezo1 in the NP with a high Pfirrmann grade (grade III~V) was significantly higher than that with a low grade. Meanwhile, the lateral radiograph revealed the local cervical curvature was significantly lower in the high grade IVDD group compared to the approximately normal group. Furthermore, the elasticity modulus in the high grade IVDD group was also clearly greater than that in the low grade IVDD group (Fig. 1).

### Histological analysis and the expression of Piezo1 and type II collage in human nucleus pulposus tissues

**Fig. 2:**
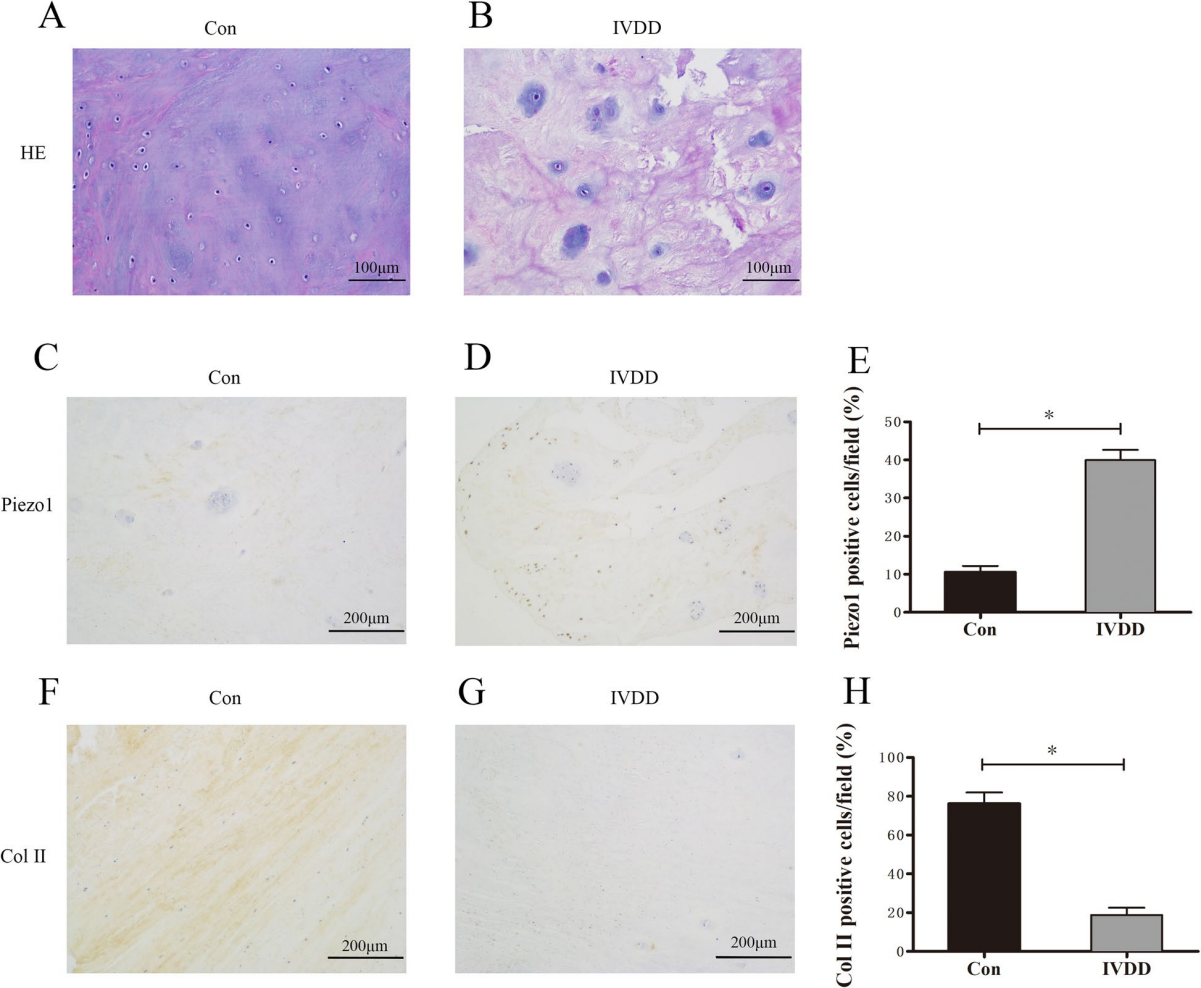
Histological analysis and the expression of Piezo1 and type II collage in human nucleus pulposus tissues. **A**, **B** Hematoxylin and eosin (H&E) staining in human NP tissues of the control or the IVDD group (200×). **C**, **D** Piezo1 immunostaining in human NP tissues of the control or the IVDD group (100×). **E** The percentage of Piezo1 positive cells to all cells in the field (%). **F**, **G** Type II collage immunostaining in human NP tissues of the control or the IVDD group (100×). **H** The percentage of Col II positive cells to all cells in the field (%). *p < 0.05

Histological assessment of NP in IVDD patients showed disorganized arrangement and reduced number of NP cells (Fig. 2A, B). The expression of Piezo1 and Col II in nucleus pulposus tissues of the control group and that of the IVDD patients were detected by immunohistochemistry. The results showed that the expression of Piezo1 had obviously increased in the NP tissues of the IVDD patients when compared to that of the control group (Fig. 2C, D). In contrast, as shown in Fig. 2F, G, the expression of Col II had significantly declined in the IVDD group when compared to that in the control group.

### Effects of mechanical stress treatment on Piezo1 and cell function in human NP cells

**Fig. 3:**
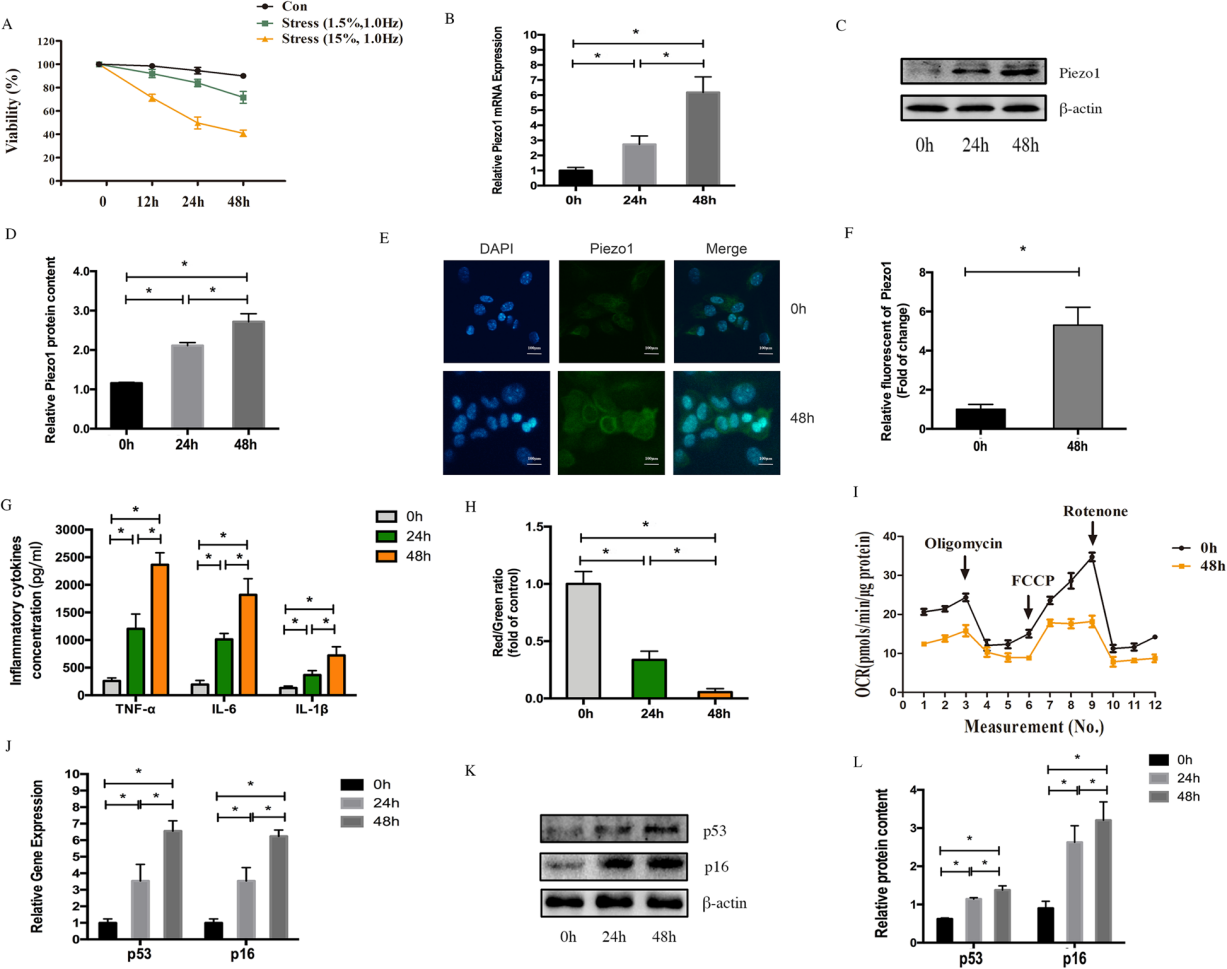
Piezo1 expression and cell function of human nucleus pulposus cells under mechanical stress. **A** NP cells received various mechanical stress treatment. Cell viability was measured by MTT assay. **B** Gene expression of Piezo1 was measured by real-time PCR. **C**, **D** Western blot analysis of Piezo1’s protein level. Total β-actin served as loading controls. **E**, **F** Immunofluorescence staining analysis of Piezo1 expression. Relative fluorescent levels of Piezo1 were measured by Image J software. **G** Concentrations of pro-inflammatory cytokines TNF-α, IL-1β, and IL-6 in supernatants were measured by ELISA. **H** Mitochondrial membrane potential was measured by JC-1 probe and flow cytometer. **I** OCR of cells were measured by Seahorse XFe96 Extracellular Flux Analyzer at basal conditions and with serial administration of oligomycin, FCCP and rotenone. **J**–**L** Gene and protein expressions of P53 and P16. Total β-actin served as loading controls. *p < 0.05. All experiments were repeated at least three times

In order to explore whether mechanical stress treatment can affect Piezo1 expression and cell function of NP cells, a mechanical compression with different intensity and/or duration was conducted on human NP cells. The effect of mechanical stress on the viability of NP cells was examined by MTT assay. As illustrated in Fig. 3A, low-intensity stress (1.5% compression rate) treatment showed little cytotoxic effect to NP cells. However, cells subjected to high-intensity stress (15% compression rate) treatment showed a significantly decreased viability in a time-dependent manner. The expression of Piezo1 was measured after high-intensity stress (15% compression rate) treatment for different durations. After a 24 h-treatment, the gene and protein levels of Piezo1 had increased, which showed a further increase after 48 h of treatment (Fig. 3B, D). The results of immunofluorescence staining also revealed that high-intensity stress treatment for 48 h clearly increased Piezo1 protein expression (Fig. 3E, F). Concurrently, we found that high-intensity stress treatment not only increased the expressions of inflammatory cytokines TNF-α, IL-6, and IL-1β but also induced mitochondrial damage by reducing mitochondrial membrane potential and oxygen consumption rate in a time-dependent manner (Fig. 3G, I). Additionally, the results of real-time PCR and western blot revealed that high-intensity stress treatment also amplified the expressions of classical senescence markers p53 and p16^INK4a^ in NP cells (Fig. 3J–L).

### Inhibition of Piezo1 alleviated mechanical stress induced imbalance of autophagy/apoptosis in human NP cells

**Fig. 4:**
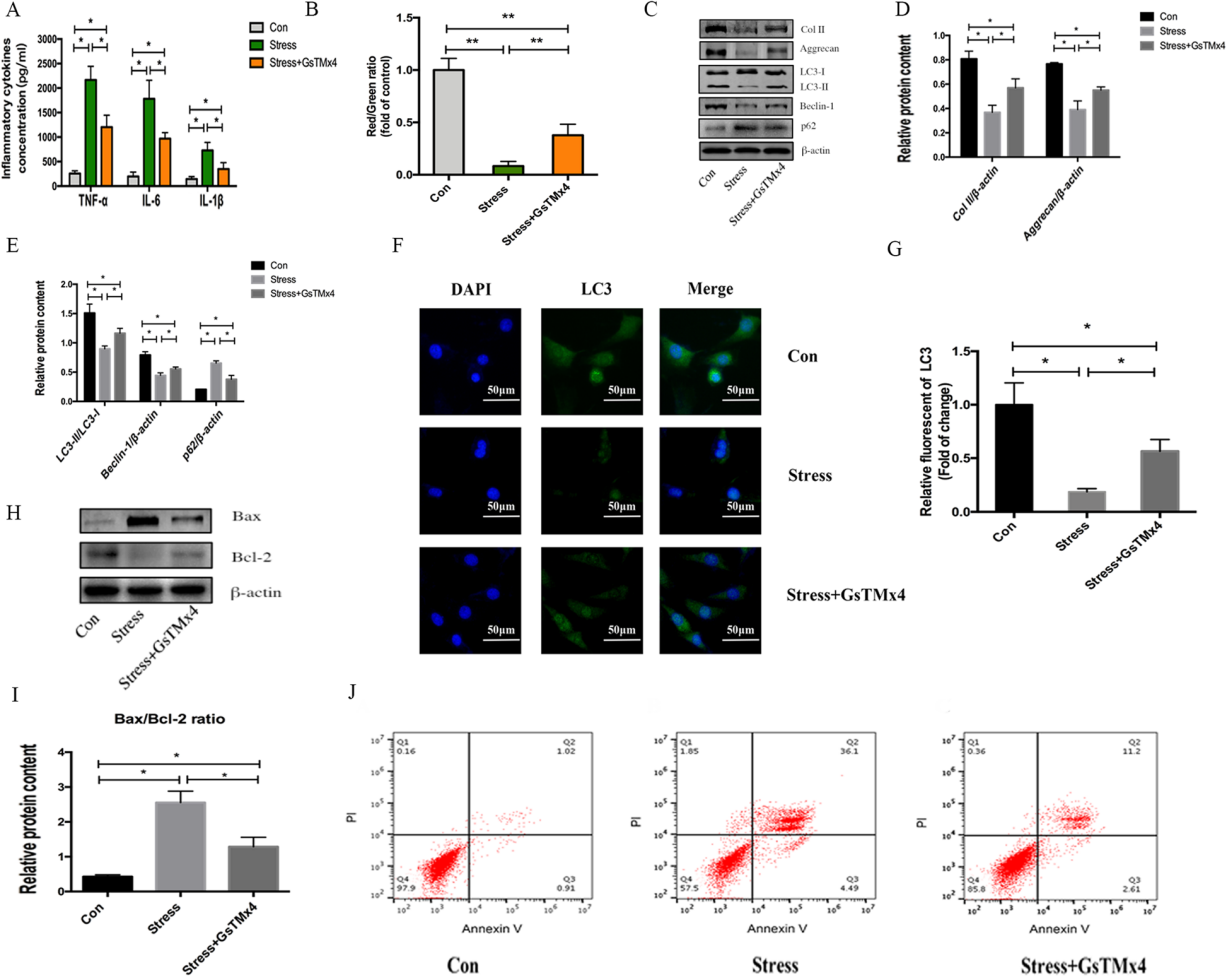
Autophagy and apoptosis related protein expressions in human nucleus pulposus cells after mechanical stress treatment. **A** Concentrations of pro-inflammatory cytokines TNF-α, IL-1β, and IL-6 in supernatants after mechanical stress treatment with/without administration of Piezo1 inhibitor GsMTx4. **B** Mitochondrial membrane potential was measured after mechanical stress treatment with/without GsMTx4. **C**–**E** Western blot analysis of protein levels of Col II, Aggrecan, LC3 II, LC3 I, Beclin-1, P62. Total β-actin served as loading controls. **F**, **G** Immunofluorescence staining analysis of LC3 expression after mechanical stress treatment with or without GsMTx4, relative fluorescent levels of LC3 were measured by Image J software. **H**, **I** Western blot analysis of Bax and Bcl-2’s protein levels. Total β-actin served as loading controls. **J** Cell apoptosis was assessed by flow cytometer with Annexin V-FITC/PI apoptosis detection kit. *p < 0.05. Data were obtained from three independent experiments

To further verify the effect of Piezo1, we used Piezo1 specific inhibitor GsMTx4 to interrupt the activation of Piezo1. As shown in Fig. 4A and Fig. 4B, GsMTx4 pre-treatment effectively reduced the expressions of pro-inflammatory cytokines TNF-α, IL-6, and IL-1β and surely recovered mitochondrial damage which were induced by mechanical stress. Meanwhile, the protein levels of Col II and Aggrecan were markedly suppressed after mechanical stress treatment but could be partially restored by GsMTx4 pre-treatment (Fig. 4C, D). Furthermore, we used western blot and LC3 visualization to detect the level of autophagy. The results in western blot revealed that mechanical stress treatment simultaneously decreased LC3II, Beclina-1 protein levels and increased P62 protein level in NP cells, indicating a decrease in the autophagy process. However, pre-treatment with GsMTx4 could reverse the expression of the proteins mentioned above (Fig. 4C, E). Similarly, the result of immunofluorescence staining showed that the LC3 level had also significantly decreased by mechanical stress treatment but was regained when pre-treated with GsMTx4 (Fig. 4F, G). The expressions of apoptosis-related proteins Bax and Bcl-2 were also detected by western blot. The ratio of protein Bax to Bcl-2 was increased by mechanical stress administration but was suppressed by inhibiting Piezo1(Fig. 4H, I). The apoptosis rate was also assessed using an Annexin V-FITC/PI apoptosis kit, which further verified that apoptosis was significantly increased in mechanical stress-treated NP cells and could be restricted when Piezo1 was inhibited (Fig. 4J).

## Discussion

**Fig. 5:**
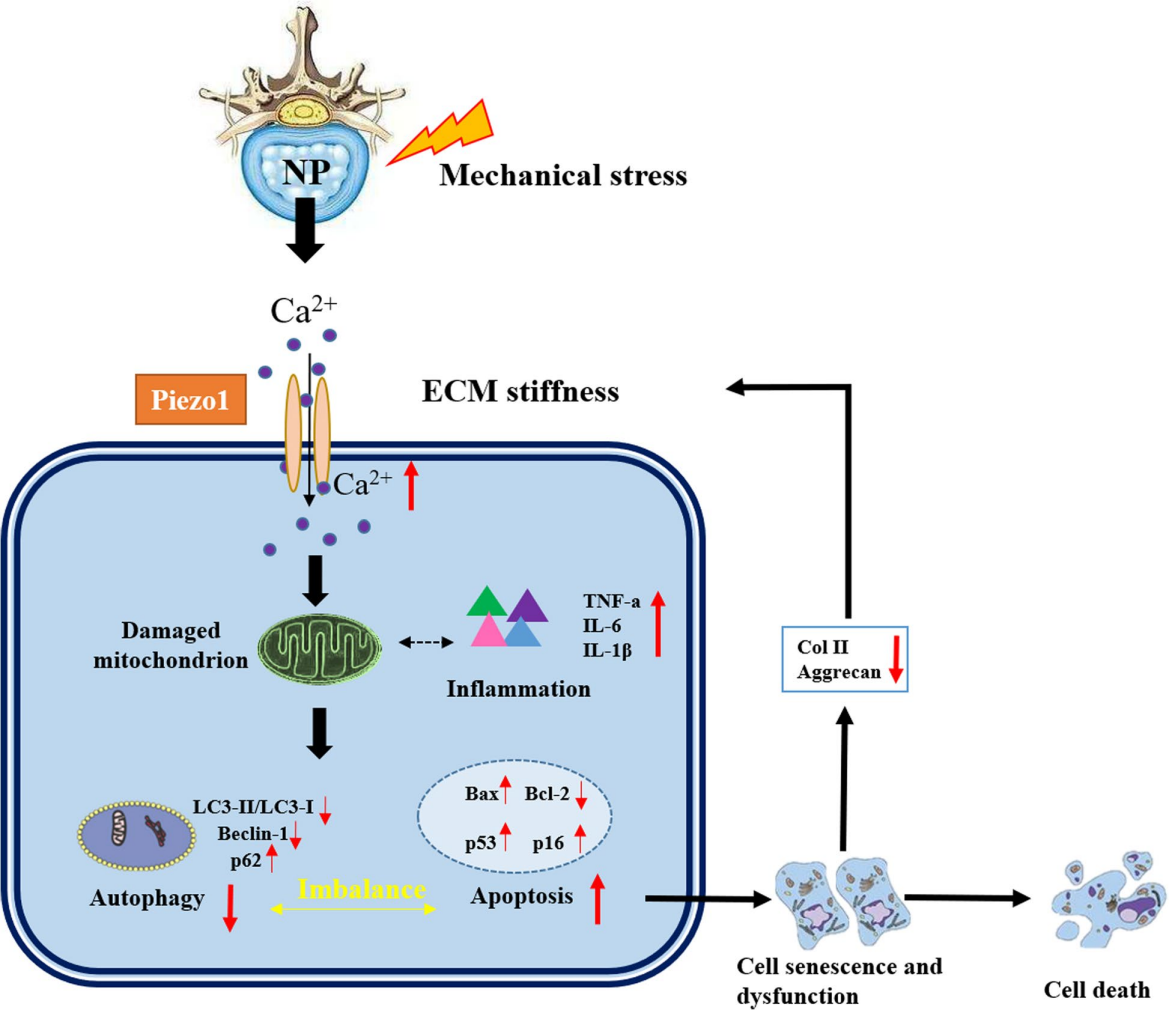
Excessive mechanical stress stimulates mechanosensitive channel protein Piezo1 overexpression in human nucleus pulposus cells, which aggravates mitochondrial damage and inflammatory response in NP cells and further triggers the reduction of autophagy and the intensification of apoptosis. The imbalance between autophagy and apoptosis may result in cell senescence and dysfunction, weaken the synthesis of collagen II and Aggrecan in NP cells, and consequently precipitate cell death and exacerbate disc degeneration

This study demonstrated that the up-regulation of the mechanosensitive ion channel Piezo1 by excessively external compressive stress can increase pro-inflammatory cytokines, and induce mitochondrial dysfunction, which may trigger the apoptosis and senescence of human NP cells via suppressing autophagy and resulting in the loss of ECM (Fig. 5).

The disc suffers complex loading such as gravitational force, varying in frequency, magnitude, and duration [17]. While the central NP transfers and distributes axial loading to the peripheral annulus fibrosus, it was well known that the rat tail bending model was utilized to simulate the pathological process of IVDD similar to sedentary lifestyle involving mechanical compression [18]. Other studies from Teraguchi et al. and Shi et al. revealed that the altered stress induced by obesity may dramatically and effortlessly increase the degenerative degrees of discs, compared with the pro-inflammatory factors [6, 19]. Additionally, intradiscal pressure was shown to be definitely higher in forward leaning from a sitting position than in relaxed sitting [20], which indicates that intradiscal pressure increases with application of compression and is dependent on the lifestyle. As previously reported, excessive mechanical stresses are found in the kyphotic areas of sagittal cervical spine when compared to the normal lordosis [21]. Moreover, the increased mechanical stress induced by kyphosis was considered to be a risk factor for the development of IVDD or disc herniation [22]. Iatridis et al. [18] concluded that chronically external compressive forces resulted in changes in mechanical properties and composition of tail discs without other disease process. Liang et al. [16] also reported that the stiffened collagen fibrils and the matrix, and increased elastic modulus were revealed in the NP of rat tail model after abnormal loading. Results in this research also demonstrated the elevated elastic modulus, up-expressed Piezo1, and reduced Col II in the degenerated human NP tissue were along with more kyphotic angle in the cervical spine, which reveals that the Piezo1 sensing the excessive stress partly attributed to poor curvature may affect the ECM homeostasis and further change the mechanical properties of NP.

Since the bimodal biosynthetic response to the characteristics of mechanical stress has been mentioned in the cartilage and disc, it is generally documented that relatively low compressive stress may result in an initial increase in synthesis of extracellular matrix, whereas excessive compressive stress can cause a visible decrease in synthesis [23]. In our in vitro research, we further confirmed that overactivated Piezo1 by excessive compression inhibits the synthesis of major parts in ECM involving the collagen II and aggrecan, similar with our histological findings. But GsMTx4, the inhibitor of Piezo1, can partly ameliorate the inhibitory effect.

Our in vitro data noted that the NP cells viability was inhibited, while Piezo1 was markedly upregulated in time-dependent patterns by excessive mechanical stress, which indicates the expression of Piezo 1 is closely related to cell apoptosis. Several published papers have also reported that the overexpression or overactivation of Piezo1 may lead to the apoptosis in a variety of cells [24–28]. Zhu et al. [29] also mentioned that Piezo1 is involved in the chondrocyte apoptosis due to abnormal stress. Despite its regular occurrence over a lifetime, it is currently uncertain if IVDD can be initiated by Piezo1 under excessive stress. As for the role of Piezo1 in the IVDD process, Sun et al. [30] reported that a single impact injury without structural impairment, similar to axial stress, can promote inflammatory response during IVDD process via Piezo1 activation. Moreover, Sun et al. [31] demonstrated Piezo1 sensitization induced by mechanical stretch also increased the NLPR3 inflammasome in NP cells. Similarly, the Piezo1 overactivation induces secretion of pro-inflammatory cytokines and mitochondrial dysfunction in NP cells suffering excessive mechanical stress in this study. A number of studies provide the evidence that Piezo1 activation stimulate the secretion of pro-inflammatory through the Ca^2+^ entry [28, 32]. Since Piezo1 is a nonselective cationic channel, the ions involving Ca^2+^, Mg^2+^, and so on, can pass into the membrane through Piezo1 [33]. In the current research, we also speculated that Ca^2+^ influx plays a crucial role in the signaling of Piezo1. Mitochondrial dysfunction, consisting of the increase of Bax and decrease of Bcl-2, promotes activation of caspase family, the key mechanism of apoptosis [34]. In the present study, we verified that excessive stress could statistically increase Bax and decrease Bcl-2 while partly reversing the effect in NP cells by GsMTx4, suggesting that the mitochondrial pathway may be involved in the apoptosis effect of Piezo1 overactivation. The cyclin-dependent kinase inhibitors, p53 and p16, were famous for the classical indicators of cellular senescence. In the current study, the upregulation of Piezo 1 enhanced the apoptosis of NP cells and the expression of p53 and p16, demonstrating that the excessive stresses promote the apoptosis and senescence of NP cells.

It has been found that apoptosis and autophagy were induced by moderate mechanical compression in NP cells [35]. Additionally, autophagy, functioned as a key adaptive `protection, was increased in NP cells under slight or moderate mechanical stress. However, autophagy had a dual role for IVDD adjusting to different stresses. Our experiments utilizing human NP cells revealed that high-intensity stress can inhibit the autophagy, leading to an increase in levels of apoptosis and aggravating NP cells to a bad condition. When we utilized the Piezo1 inhibitor to promote autophagy, NP cells seemed to survive better.

Undeniably, some main limitations existed in the current study. First, the animal model imitating the excessive mechanical compression is absent due to lack of appropriately stable measurements of external loadings. Moreover, the mechanism through which Piezo1 inhibits the autophagy is not yet adequately elucidated. Besides, the response of Piezo1 to slight or moderate stress was not clarified.

## Conclusions

In summary, our data provide the evidence that Piezo1 upregulation under excessive compression can promote the apoptosis and senescence of NP cells, secrete pro-inflammatory cytokines, and reduce the synthesis of ECM by mitochondrial dysfunction and the suppression of autophagy in NP, yet, the inhibition of Piezo1 can partly alleviate these effects. These findings might shed novel light on mechanobiological pathogenesis of IVDD and contribute to the search of potential methods for the prevention and therapy of IVDD.

## Data Availability

The datasets used and/or analyzed in the current study are available from the corresponding author on reasonable request.

## Abbreviations

IVDD: Intervertebral disc degeneration
NP: Nucleus pulpous
ECM: Extracellular matrix
MMP: Mitochondrial membrane potential
OCR: Oxygen consumption rate
